# Short-term Western-style diet negatively impacts reproductive outcomes in primates

**DOI:** 10.1172/jci.insight.138312

**Published:** 2021-02-22

**Authors:** Sweta Ravisankar, Alison Y. Ting, Melinda J. Murphy, Nash Redmayne, Dorothy Wang, Carrie A. McArthur, Diana L. Takahashi, Paul Kievit, Shawn L. Chavez, Jon D. Hennebold

**Affiliations:** 1 Department of Cell, Developmental & Cancer Biology, Graduate Program in Molecular & Cellular Biosciences, Oregon Health & Science University School of Medicine, Portland, Oregon, USA.; 2 Division of Reproductive & Developmental Sciences, Oregon National Primate Research Center, Beaverton, Oregon, USA.; 3 21st Century Medicine Inc., Fontana, California, USA.; 4 Division of Cardiometabolic Health, Oregon National Primate Research Center, Beaverton, Oregon, USA.; 5 Department of Molecular & Medical Genetics, Oregon Health & Science University School of Medicine, Portland, Oregon, USA.; 6 Department of Obstetrics & Gynecology, Oregon Health & Science University School of Medicine, Portland, Oregon, USA.

**Keywords:** Metabolism, Reproductive Biology, Embryonic development, Fertility, Obesity

## Abstract

A maternal Western-style diet (WSD) is associated with poor reproductive outcomes, but whether this is from the diet itself or underlying metabolic dysfunction is unknown. Here, we performed a longitudinal study using regularly cycling female rhesus macaques (*n* = 10) that underwent 2 consecutive in vitro fertilization (IVF) cycles, one while consuming a low-fat diet and another 6–8 months after consuming a high-fat WSD. Metabolic data were collected from the females prior to each IVF cycle. Follicular fluid (FF) and oocytes were assessed for cytokine/steroid levels and IVF potential, respectively. Although transition to a WSD led to weight gain and increased body fat, no difference in insulin levels was observed. A significant decrease in IL-1RA concentration and the ratio of cortisol/cortisone was detected in FF after WSD intake. Despite an increased probability of isolating mature oocytes, a 44% reduction in blastocyst number was observed with WSD consumption, and time-lapse imaging revealed delayed mitotic timing and multipolar divisions. RNA sequencing of blastocysts demonstrated dysregulation of genes involved in RNA binding, protein channel activity, mitochondrial function and pluripotency versus cell differentiation after WSD consumption. Thus, short-term WSD consumption promotes a proinflammatory intrafollicular microenvironment that is associated with impaired preimplantation development in the absence of large-scale metabolic changes.

## Introduction

Obesity is often associated with various physiological, metabolic, and psychological diseases and disorders, including hypertension, diabetes, arthritis, and depression, in both men and women (1–6). There is also a known correlation between maternal/paternal obesity and infertility or subfertility in rodents and humans (7–9). Indeed, overweight/obese women are more likely to experience reproductive issues such as menstrual irregularities (10), decreased chances of conception, and defects in placentation during pregnancy compared with normal-weight women (11, 12). However, whether the manifestations of maternal obesity are due to abnormal ovarian follicular development, interruption of the hypothalamic-pituitary-ovarian (HPO) axis that regulates ovulation, and/or changes in endometrial receptivity is unclear (10, 13).

In obese women undergoing infertility treatment, findings of unresponsiveness or a delayed response to controlled ovarian stimulation (COS) by administration of exogenous gonadotropins (8, 14–16), as well as poor in vitro fertilization (IVF) outcomes (17–19), suggests that the ovarian follicular microenvironment can be altered with obesity. This conclusion is consistent with other IVF studies, whereby increased pregnancy failure rates in obese women returned to normal if donor oocytes were used (20). Additional evidence is provided by rodent studies showing that a highly obesogenic diet caused adverse effects on murine oocyte quality and metabolism, producing mature metaphase II (MII) oocytes with chromosomal abnormalities (7, 21). Upon fertilization of these oocytes, increased embryo degradation and delayed developmental progression distinct from meiotic aneuploidy was also observed, which indicated further effects on preimplantation development (7). Transfer of maternal high-fat diet mouse blastocysts into a nonobese uterine environment still resulted in brain development defects, fetal growth retardation, and embryonic loss (7). Thus, findings of abnormal postimplantation development in these rodent studies was likely due to deficiencies in oocytes or other maternal factors rather than the uterine microenvironment (7, 22). The identity of these factors and at which stage of preimplantation development the defects occurred, however, remains to be defined.

Besides the precise contributors to embryo loss in obesity, it is also unclear, especially in women, if the negative effect on reproductive processes is due to the diet itself or the subsequent development of metabolic dysfunction. Moreover, it is difficult to distinguish these possibilities with human studies due to obvious ethical and technical limitations, as well as the considerable challenge in controlling for environmental and dietary factors that could influence reproductive outcomes. Additionally, while many studies investigated the effects of Western-style diet (WSD) utilizing rodent models, they are generally sacrificed in order to obtain oocytes or embryos, which precludes longitudinal analyses. Here, we conducted a longitudinal study, using a cohort of female rhesus macaques, that allowed us to assess the ovarian follicular microenvironment and IVF outcomes under a tightly controlled switch from a standard chow diet (SCD) to a WSD. Each rhesus macaque female served as its own experimental control such that short-term diet-induced effects on primate ovarian follicle, oocyte, and preimplantation embryo development could be assessed directly without the complicating influences of abnormal insulin homeostasis. Given similarities in reproductive physiology, response to hormonal stimulation, typical oocyte yields, and blastocyst formation rates shared between women and female rhesus macaques, our findings are translationally relevant to obese women undergoing infertility treatment in IVF clinics (23, 24).

## Results

### The effects of maternal short-term WSD intake on metabolic parameters.

**Figure 1:**
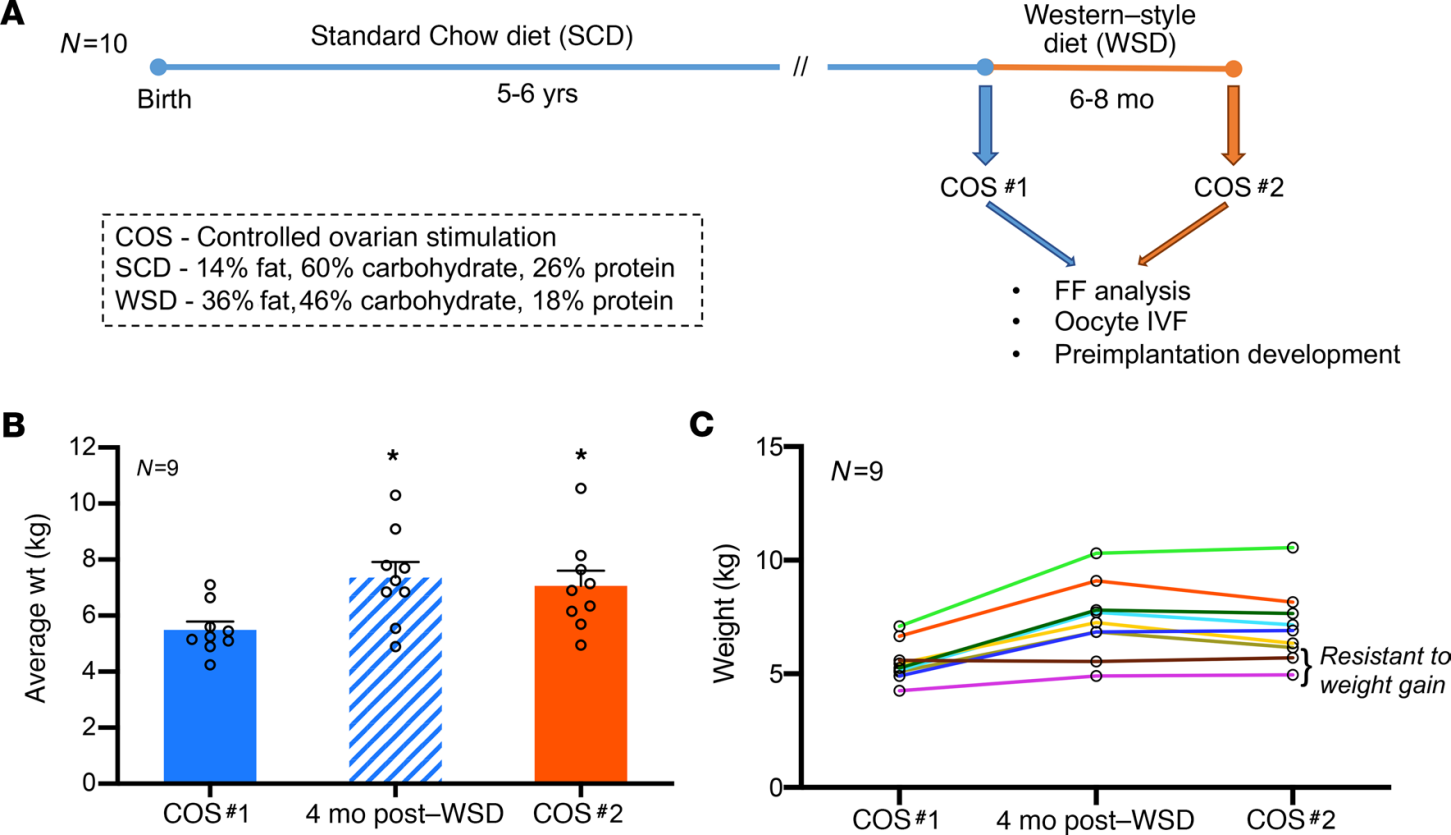
Longitudinal study to determine the effects of short-term maternal WSD intake on reproductive outcomes. (**A**) A cohort of female rhesus macaques (*n* = 10) consuming SCD since birth underwent a baseline ovarian stimulation protocol (COS #1), which was then followed by 6–8 months of high-fat WSD consumption and a second stimulation protocol (COS #2). FF and oocytes were collected from each COS to assess the intrafollicular environment and post-IVF embryo development, respectively. (**B**) A significant difference (*P < 0.05; ± SEM) was observed in the average weight of the female rhesus macaques measured after COS #1 while consuming SCD compared with 4 months after WSD consumption and after COS #2 (6–8 months after WSD consumption). (**C**) Individual weight gain comparisons revealed the resistance to weight gain displayed by 2 females. One female who was nonrespondent to COS #2, but still gained weight after being switched to a WSD, was excluded from the analysis for **B** and **C**. Data were collected from all the females that underwent both COS cycles (*n* = 9), and statistical significance was analyzed by 1-way ANOVA and a post-hoc *t* test comparison.

**Figure 2:**
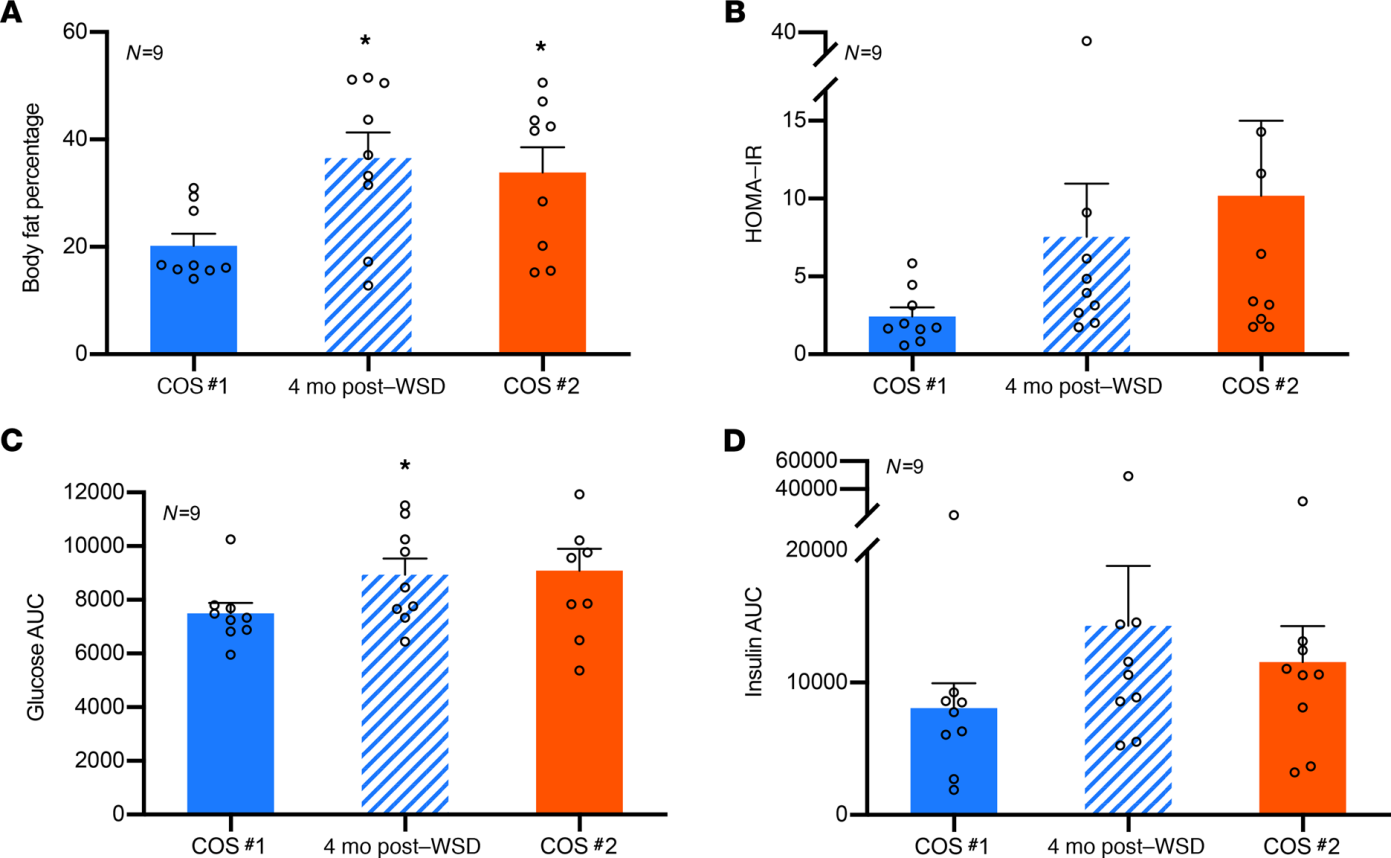
Short-term WSD consumption by female rhesus macaques does not cause significant changes in insulin resistance. (**A**–**D**)Metabolic parameters, including body fat percentage (**A**), HOMA-IR (**B**), glucose AUC (**C**), and insulin AUC (**D**) were measured after COS #1 while consuming SCD, 4 months after WSD consumption, and after COS #2 (6–8 months after WSD consumption). Percent body fat was significantly increased (*P < 0.05, ± SEM) compared with COS #1 at 4 months after WSD and after COS #2. Glucose levels were significantly (*P < 0.05, ± SEM) greater only at 4 months after WSD relative to COS #1. Data were collected from all the females that underwent both COS cycles (*n* = 9) and statistical significance was analyzed by 1-way ANOVA and a post-hoc *t* test comparison.

Ten regularly cycling female rhesus macaques of young maternal age (5–6 years old) and average body weight (5–7 kg) were selected for this longitudinal study (Figure 1A). Each female consumed a SCD (15% fat, 59% carbohydrate, 26% protein) once weaned and underwent an initial COS cycle (COS #1). The females were then switched to a high-fat WSD (36% fat, 46% carbohydrate, 18% protein) for 6–8 months and underwent a second COS cycle (COS #2). Weight, body fat percentage, circulating glucose and insulin levels, and homeostatic model assessment of insulin resistance (HOMA-IR) were measured 1–3 months after COS #1 while consuming a SCD, 4 months after transitioning to a WSD, and 1 month after COS #2 (approximately 6–8 months after beginning the WSD). Because 1 female did not undergo COS #2 (detailed below), the average weight for the remaining 9 female rhesus macaques was assessed during the 3 time periods. Overall weight gain (Figure 1B) significantly increased (*P* < 0.05) as early as 4 months after WSD consumption, which was retained 6–8 months later. When the weight of each female was analyzed separately, 2 animals appeared to be resistant to weight gain (Figure 1C), which is typical of nonhuman primate diet studies (25). Similar to weight gain, percent body fat (Figure 2A) showed a statistically significant (*P* < 0.05) increase as early as 4 months after WSD consumption and was maintained 6–8 months later. Although there was a significant difference in glucose levels 4 months after WSD, no significant differences in metabolic parameter measurements were observed after COS #1 and after COS #2, which included HOMA-IR (Figure 2B), glucose (Figure 2C), and insulin (Figure 2D) levels. Thus, while the majority of females exhibited significant weight gain and increased body fat from short-term WSD consumption, glucose levels and insulin secretion were not significantly different between COS #1 and COS #2.

### Responsiveness to gonadotropin stimulation before and after WSD consumption.

All 10 rhesus macaque females responded positively to exogenous gonadotropin administration during COS #1 based on increases in circulating E2 levels that were observed over the course of the stimulation protocol. However, 2 of the 10 females failed to respond to hormonal stimulation after consuming the WSD (COS #2). It is important to note that the 2 nonresponders were different from the 2 rhesus macaque females that were resistant to weight gain. In 1 of the 2 non-respondent females, E2 levels never increased above 200 pg/mL on day 3 or day 4 of the COS #2 protocol, but the follicular aspiration proceeded and only a small number of oocytes was obtained. In the other nonrespondent female, estradiol levels never increased above baseline and the post-WSD COS (COS #2) was terminated. We note that these results are in accordance with previous human studies, whereby unresponsiveness or a delayed response to exogenous gonadotropin administration was reported in obese women undergoing IVF (8, 14–16).

### Impact of short-term WSD consumption on intrafollicular steroid and cytokine levels.

**Figure 3:**
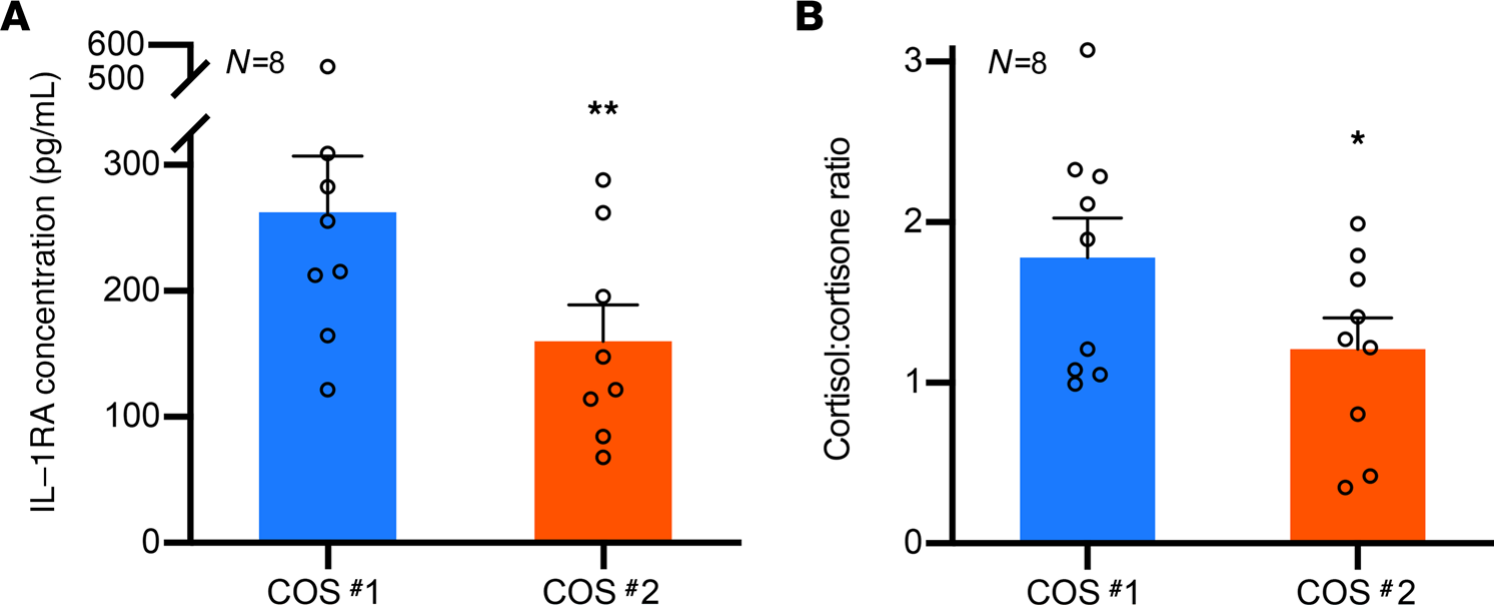
The cytokine and steroid milieu in rhesus macaque ovarian follicles are affected by short-term WSD intake. Cytokines and steroids were measured in the FF after COS #1, while consuming SCD and after COS #2 (6–8 months after WSD consumption) by the Luminex 29-plex platform and LC/MS, respectively. (**A** and **B**) There was a significant decrease in the concentration of IL-1RA (**A**) (**P < 0.01, ± SEM) and the ratio of cortisol/cortisone (**B**) (*P < 0.05, ± SEM) after 6–8 months of WSD consumption. Only females that responded to the COS from which a sufficient volume of FF was collected were included in this analysis (*n* = 8), and a paired *t* test was performed to assess statistical significance.

Our next objective was to assess the intrafollicular steroid and cytokine milieu of ovarian follicles in the rhesus macaque females at the time of oocyte aspiration. This was accomplished by obtaining follicular fluid (FF) samples from each female both before and after WSD consumption and analyzing the concentrations of cytokines and steroids by cytokine array and liquid chromatography–tandem mass spectrometry (LC-MS/MS), respectively. Of the 29 cytokines analyzed (Supplemental Table 1; supplemental material available online with this article; https://doi.org/10.1172/jci.insight.138312DS1), a significant decrease (*P* < 0.01) in the concentration of IL-1 receptor antagonist (IL-1RA) was observed in FF after WSD consumption (Figure 3A). A significant reduction (*P* < 0.05) in the level of IL-2 was also detected with WSD intake (data not shown). From the 7 steroids assessed (Supplemental Table 2), there was an increase in the average cortisone concentration, with a corresponding decrease in the average cortisol concentration, in the FF after WSD consumption, resulting in a significantly decreased (*P* < 0.05) cortisol/cortisone ratio (Figure 3B). Because both IL-1RA and cortisol have antiinflammatory properties (26, 27), these results suggest that WSD consumption led to an increased inflammatory state in primate ovarian follicles.

### Correlation between short-term WSD consumption and oocyte maturity.

To further assess the effects of WSD consumption on the intrafollicular microenvironment, oocytes were obtained after each COS from rhesus macaque females both before and after WSD consumption. A total of 527 oocytes were collected from the 10 females during COS #1, with an average of 53 oocytes per female, while 399 oocytes were obtained from 9 of the females during COS #2, with an average of 44 oocytes per animal. Aspirated oocytes were classified as either a mature metaphase II (MII) oocyte, maturing MI oocyte, immature germinal vesicle (GV) oocyte, or degenerated oocyte. The odds of isolating a GV oocyte from a female on the WSD was 0.611 times the odds of when the female was consuming the SCD (95% CI, 0.402–0.929 times; *P* < 0.05). In contrast, the odds of obtaining MII oocytes was higher, but not statistically significant, after WSD consumption. Thus, aspirated oocytes were less likely to be at the GV stage and more likely to be in the MII stage of development when the female was consuming the WSD.

### Preimplantation embryo fate following SCD versus WSD consumption.

**Figure 4:**
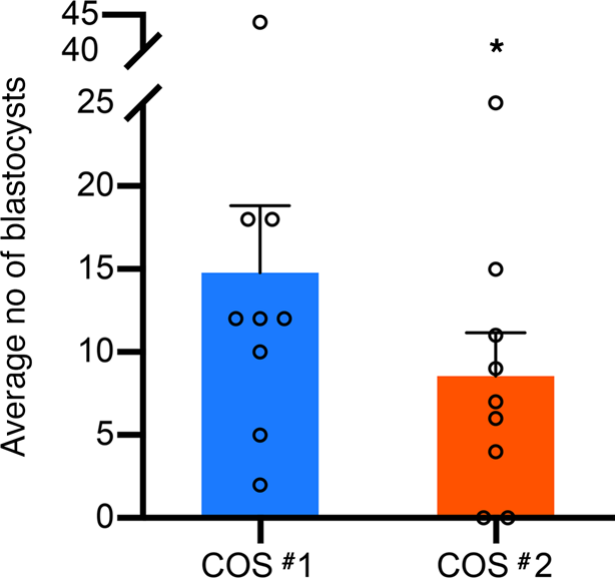
Short-term WSD consumption is associated with reduced blastocyst formation. The number of blastocysts formed after WSD consumption decreased by 44% relative to that formed after SCD intake, and a statistically significant difference (*P < 0.05, ± SEM) was detected when the average number of blastocysts formed per female rhesus macaque was analyzed. Data were collected from all the females that underwent both COS cycles (*n* = 9), and a paired *t* test, along with the logistic mixed effects regression models with random intercept, was used to determine statistical significance.

The isolated oocytes were fertilized and IVF-developmental outcomes were compared between COS #1 and COS #2. While the average fertilization rate after SCD versus after WSD (68% and 73%, respectively) was not statistically different, we did observe a significant decrease (*P* < 0.05) in the average number of blastocysts formed per female (Figure 4). In total, we obtained 138 blastocysts from the females on the SCD and only 77 blastocysts after transitioning animals to the WSD, resulting in a 44% reduction in the number of blastocysts. Statistical testing determined that the odds of forming a blastocyst from a female after WSD consumption was reduced, with the odds being 0.673 times that of animals consuming a WSD compared with when the female was receiving a SCD (95% CI, 0.485–0.935 times). FF analysis revealed that the ability of the resident oocyte to form a blastocyst following IVF was also negatively correlated with a reduced cortisol/cortisone ratio after WSD intake (*R* = –0.66; *P* < 0.05).

### WSD intake is associated with delayed mitotic timing and multipolar divisions in embryos.

**Figure 5:**
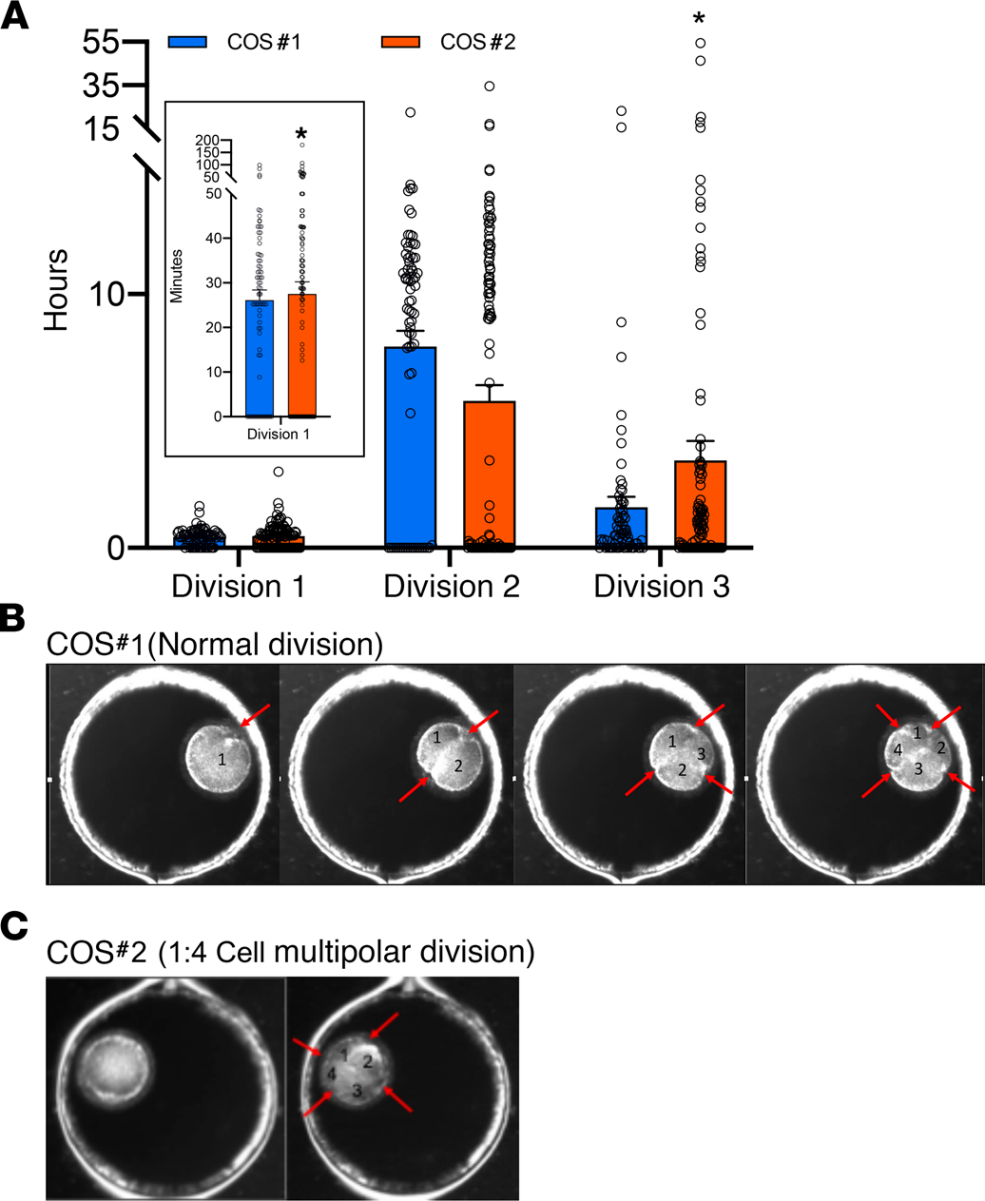
Delayed mitotic timing and multipolar divisions were observed in WSD preimplantation embryos. The time intervals between the appearance of the first cleavage furrow to the end of the first cytokinesis, the beginning of the second mitotic division, and the start of the third mitotic division were measured during preimplantation development until the embryo arrested or formed a blastocyst in COS #1 versus COS #2. (**A**) These measurements were averaged (data shown as ± SD) among 5 independent reviewers and the first and third division were significantly longer (*P < 0.05) in the embryos obtained after WSD consumption relative to after SCD intake. (**B**) Individual image frames from TLM videos of a representative SCD embryo showing normal bipolar divisions up to the 4-cell stage. (**C**) Similar imaging of a representative WSD embryo revealed a 1:4 cell multipolar division at the zygote stage. The cleavage furrows are denoted by red arrows in **B** and **C**. The corresponding full-length TLM movies of these 2 embryos can be viewed in Supplemental Video 1 (after SCD) and Supplemental Video 2 (after WSD). A paired *t* test, along with the logistic mixed effects regression models with random intercept, was used to determine statistical significance.

To determine if there were morphological or other characteristics that might explain the lower number of blastocysts formed following WSD consumption, the development of fertilized SCD versus WSD oocytes was monitored by time-lapse imaging up to the blastocyst stage. A total of 174 time-lapse videos following either pre- or post-WSD intake were analyzed, and the timing of initial mitotic divisions were measured based on their predictive value of blastocyst formation in human embryos (28, 29). Of the first 3 mitoses, the duration of the first mitotic division and time interval between the second and third mitotic division was 0.15 ± 0.03 and 3.25 ± 0.07 hours longer (*P* < 0.05) in post-WSD compared with post-SCD intake, respectively (Figure 5A). A higher incidence of cellular fragmentation was also observed in the embryos obtained from females following WSD consumption, but this was not statistically significant. While normal bipolar divisions more often occurred in SCD embryos (Figure 5B and Supplemental Video 1), a higher frequency of multipolar divisions was observed in WSD embryos at the zygote stage or later in development (Figure 5C and Supplemental Video 2), albeit also statistically insignificant. We note that delayed mitotic timing and multipolar divisions were detected even in embryos from the females that were resistant to weight gain. By combining these results, we determined that the odds of observing a multipolar division in an embryo after WSD consumption was 1.617 times the odds of an abnormal division occurring in an embryo from a female receiving a SCD. Although there was sizeable variability in blastocyst formation (95% CI, 0.577–4.687 times), the WSD embryos were more likely to arrest at the cleavage stage than progress in development compared with SCD-derived embryos.

### Differential gene expression in blastocysts following SCD versus WSD consumption.

**Figure 6:**
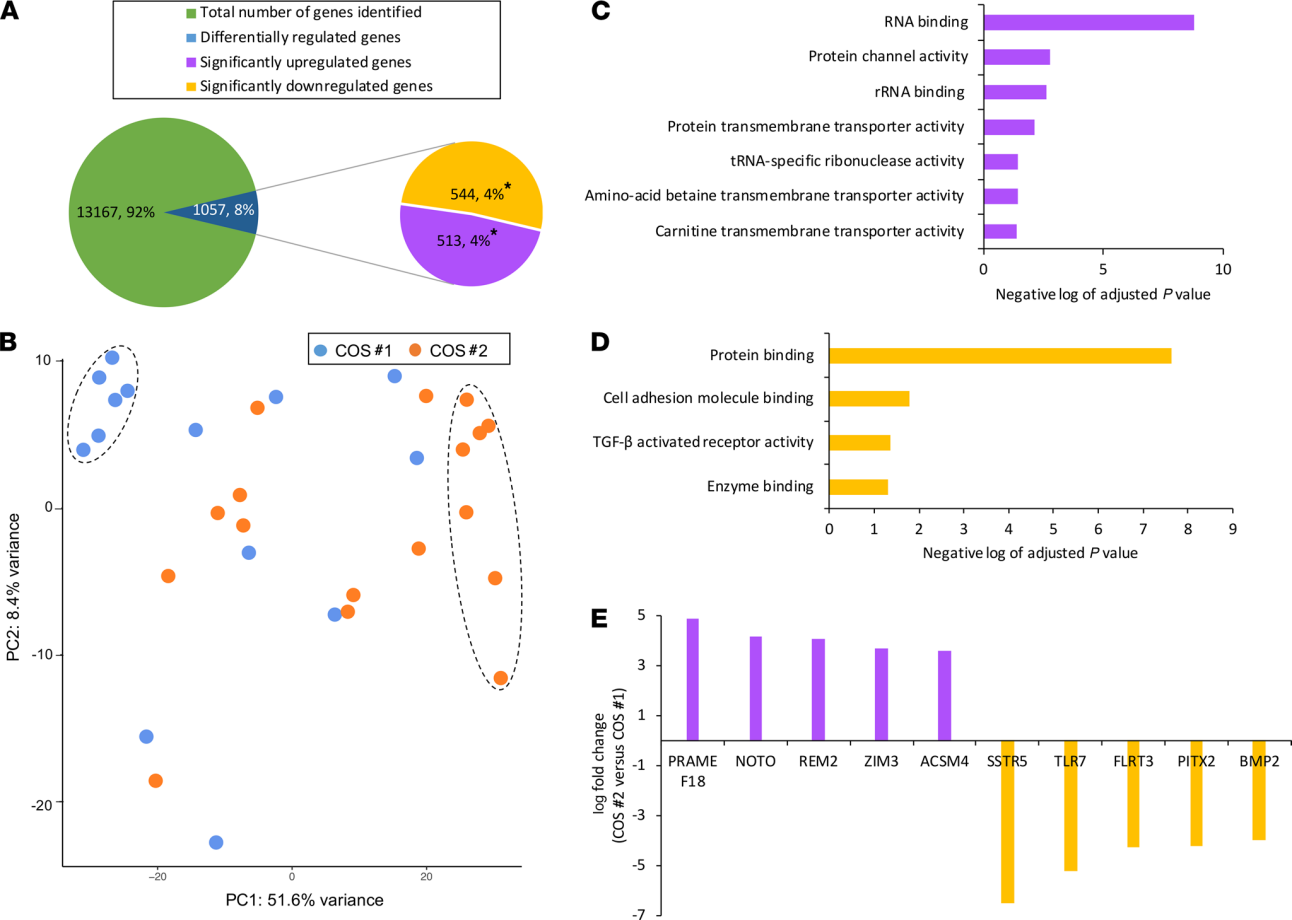
RNA-seq analysis of blastocysts reveals that short-term WSD consumption leads to changes in gene expression. (**A**) A total of 13,167 genes were identified from the RNA-seq analysis of 31 blastocysts (*n* = 14 for SCD; *n* = 17 for WSD), out of which 1057 genes (5%) were differentially expressed. Of the differentially regulated genes (DEGs), 544 (~4% of the total number of genes and 51% of the DEGs) were significantly downregulated and 513 genes (~4% of the total number of genes and 49% of the DEGs) were significantly upregulated in the blastocysts obtained from animals receiving a WSD versus the blastocysts that were obtained from animals fed a SCD (*P < 0.05). (**B**) Principal component analysis (PCA) revealed 2 distinct populations of blastocysts representative of the samples obtained from COS #1 and COS #2 and denoted by dashed ovals. (**C** and **D**) The top gene ontology (GO) terms for molecular functions of the significantly upregulated (**C**) and significantly downregulated (**D**) genes are represented. The *x* axis is the negative log of the adjusted *P* value. (**E**) From the list of significantly DEGs, the top upregulated (16- to 32-fold) and downregulated (16- to 64-fold) genes in WSD blastocysts are shown. The *x* axis indicates the gene names, and the *y* axis represents the log_2_ fold change in gene expression. For **C**–**E**, the purple bars denote the upregulated genes, and the yellow bars indicate the downregulated genes. RNA-seq data *P* values were adjusted for multiple comparisons with the Benjamini-Hochberg method.

Of the 215 total blastocysts obtained from the rhesus macaque females either before or after WSD intake, 33 individual blastocysts (*n* = 15 for SCD; *n* = 18 for WSD) were used for RNA sequencing (RNA-seq) (Supplemental Table 3). To ensure that the same animals were predominantly represented and to avoid individual heterogeneity, blastocysts from 5 females after SCD and 7 females after WSD, including 5 of the same females as after SCD, were used in the analysis. After sequence alignment and quality assessment of the RNA-seq data, we determined that 1 sample from the SCD group and another from the WSD group had lower gene counts than expected and appeared as outliers when compared with the other embryos represented by Euclidean distance clustering (Supplemental Figure 1). Therefore, these 2 samples were removed from further examination, and the remaining 31 blastocysts (*n* = 14 for SCD; *n* = 17 for WSD) were carried forward for differential gene expression analysis. We identified 13,167 genes, 1057 (8%) of which were differentially regulated between the blastocysts obtained from the 2 treatment groups. An almost equal percentage of the genes was significantly upregulated (513 genes; approximately 49%) or downregulated (544 genes; approximately 51%) in blastocysts formed after WSD consumption versus those following SCD intake (Figure 6A, P** < 0.05). Principal component analysis (PCA) revealed a subset of blastocysts from COS #1 (*n* = 6) and COS #2 (*n* = 7) that each clustered together in 1 of 2 sub-groups based on diet (Figure 6B), while the other blastocysts did not separate according to SCD versus WSD or which female they came from.

### WSD consumption impacts biological processes crucial to preimplantation development.

Gene ontology (GO) and pathway analyses were performed to identify the biological processes and molecular functions most associated with WSD-induced gene regulation. The upregulated genes were enriched in functions such as RNA binding, protein channel activity, tRNA-specific ribonuclease activity, and transmembrane amino acid betaine and carnitine transporter activity (Figure 6C). Of the upregulated genes in WSD blastocysts, the highest ranked belonged to diverse biological processes, including retinoic acid metabolism, GTP binding and GTPase activity, maternal imprinting, and pluripotency maintenance (30–32). Additional biological processes that other upregulated genes belonged to were mostly involved in mitochondrial translation, which suggested that the blastocysts had altered mitochondrial function after WSD intake. Following pathway analysis of the downregulated genes in blastocysts, protein binding, cell adhesion molecule binding, TGFB receptor activity, and enzyme binding were identified as the molecular processes associated with consuming a WSD (Figure 6D). We note that these pathways are involved in critical biological processes during embryogenesis such as activation or stimulation of cell migration, differentiation, growth factor signaling, and tissue development (33, 34).

Among the 513 genes that were significantly upregulated in WSD compared with SCD blastocysts, the top genes ranked according to fold-change were PRAME family member 18 (*PRAMEF18*; 20-fold), notochord homeobox (*NOTO*; 15-fold), GEM like GTPase 2 (*REM2*; 13-fold), acyl-CoA synthetase medium chain family member 4 (*ACSM4*; 12-fold), and killer cell lectin like receptor G2 (*KLRG2*; 11-fold), as shown in Figure 6E. In addition to the top upregulated genes, pathways related to mitochondrial translation elongation, translation termination, and mitochondrial transport (*PTCD3*, *TIMM17A*, *TOMM22*, *TOMM40*, *TOMM 20*, *MRPL46*, *MRPL45*, *MRPL38*, *MRPL37*, *MRPS35*, *MRPS16*, *MRPS25*, *SLC25A19*, *SLC25A10*, *SLC25A20*, and *SLC25A2*) were also upregulated in WSD blastocysts — but to a lesser extent. The most drastically downregulated gene in WSD blastocysts was the somatostatin receptor 5 (*SSTR5*), with an 88-fold lower expression in blastocysts after WSD compared with the SCD. This was accompanied by a significant decrease in the expression of toll like receptor 7 (*TLR7*; 37-fold), zinc finger protein 750 (*ZNF750*; 28-fold), DISC1 scaffold protein (*DISC1*; 21-fold), and fibronectin leucine rich transmembrane protein 3 (*FLRT3*; 19-fold) in WSD versus SCD blastocysts (Figure 6C). Besides these genes, factors and pathways associated with BMP signaling and mesodermal commitment (*HAND1*, *FOXA2*, *TRIM71*, *BMPR2*, *BMPR1A*, *BMP2*, and *FGFR1*), focal adhesion (*IGF1*, *COL4A2*, *COL4A1*, *PAK1*, and *ACTN1*), and endoderm differentiation (*CTNNB1*, *FOXA2*, *TRIM71*, and *BMPR1A*) were significantly reduced in blastocysts after WSD intake. Combined with findings of increased expression of pluripotency genes in WSD blastocysts, this suggested that the WSD induced dysregulation of downstream cell differentiation pathways important for peri-implantation development.

## Discussion

Maternal obesity and its negative impact on IVF treatments has been reported in both humans and rodents (7, 35, 36), but it was unclear if these effects were due to the diet itself or the subsequent development of metabolic dysfunction. To our knowledge, we present the first longitudinal study to assess the consequences of a short-term WSD consumption on the ovarian microenvironment and preimplantation development in the translationally relevant rhesus macaque model. With the exception of 2 females that were resistant to weight gain, we observed an overall increase in body weight and body fat percentage as early as 4 months after WSD intake that was preserved after 6–8 months of consumption. There were also 2 other females who were less responsive or nonresponsive to ovarian stimulation, which is in concordance with previously reported studies indicating that obese women undergoing assisted reproduction for infertility treatment not only often require extended periods of gonadotropin stimulation, but also required increased amounts of gonadotropin compared with normal-weight women (8, 15, 16). Obese women also experience a higher incidence of IVF cycle cancellation (14), and weight loss has been reported to improve the detrimental effects of obesity on reproductive potential in these patients (8, 10). Despite the weight gain and increase in percent body fat, we did not observe significant changes in insulin resistance, which were reported in rodent studies investigating high-fat diet effects on oocyte competency (10). There are a number of factors that could explain the discrepancy between these rodent studies and our findings, including the duration of WSD consumption (37–39), differences in the percentage of calories from fat (40), and the idea that a certain percentage of the nonhuman primate population is resistant to the metabolic effects of the high-fat diet (37). While WSD consumption did not significantly alter insulin and glucose levels after COS #2, it is possible that other processes were altered that could have an effect on the follicle and the resident oocyte, including increased local or circulating inflammatory factors or adipokines (41–43). We would also like to note that statistical analyses did not yield any significant differences between oocyte number, blastocyst formation, or RNA-seq data for the females that gained weight versus those that did not.

A disturbance in the intrafollicular milieu from the WSD was demonstrated by a decrease in IL-1RA concentration in the FF of ovarian follicles. As an antagonist to the IL-1R, IL-1RA inhibits downstream signaling of the proinflammatory cytokine, IL-1, upon receptor binding (26). This suggests that the maternal high-fat WSD promotes heightened inflammatory activity in the follicle that, in turn, may induce aberrant developmental programming in the offspring produced from the developing oocyte as previously described (44). Further support for alterations in the inflammatory potential of the follicle is supported by a decrease in the ratio of cortisol/cortisone in the FF since cortisol is a potent antiinflammatory steroid hormone (27, 45) that mediates its effects through binding and activating the glucocorticoid receptor (46). Cortisone, in contrast, is biologically inactive due to its minimal glucocorticoid receptor binding capacity. Indeed, high concentrations of cortisol or elevated cortisol/cortisone ratios in the FF of periovulatory follicles was shown to correlate with human IVF outcomes in previous studies (47–49). From multiple studies across species, FF and its constituents were also reported to influence oocyte competency, or the ovum’s ability to undergo meiotic maturation, fertilization, and preimplantation development (50–58). Thus, we suggest that the loss of antiinflammatory factors within the intrafollicular microenvironment produced as a result of a WSD limits the potential of the resident oocyte to yield a blastocyst following IVF. WSD consumption also resulted in a reduction in FF IL-2 levels (data not shown), which is known to be produced primarily by activated CD4^+^ T cells in secondary lymphoid organs, where it promotes T cell proliferation and inflammatory processes (59). IL-2 has been detected in the FF of women after gonadotropin stimulation and exhibits variable effects on steroidogenesis in mural granulosa cells and luteal cells (60–63). Increased synthesis of IL-2 by mural granulosa cells obtained from women with ovarian hyperstimulation syndrome has also been reported (64), but the significance of IL-2 in the ovarian follicle remains to be determined.

Despite the decreased odds of aspirating an immature GV oocyte and a higher probability of obtaining a MII oocyte with short-term WSD consumption, reduced blastocyst yields were observed in post-WSD embryos. The isolation of predominantly mature oocytes was somewhat unexpected, since we previously reported that long-term (3–4 years) WSD consumption in rhesus macaque females led to the retrieval of 33%–43% degenerated oocytes aspirated from the naturally selected dominant follicle prior to ovulation (65). While aneuploidy was not directly assessed, given that aneuploid embryos often arrest and/or are developmentally delayed (66), this could also be a cause for the reduced blastocyst formation after WSD consumption as previously shown in a high-fat diet rodent model (7). Indeed, time-lapse imaging demonstrated that the duration of the first mitotic division and time interval between the second and third mitotic division was significantly longer in embryos after WSD than after SCD intake. It also revealed a greater incidence of multipolar divisions in WSD embryos, which are often associated with aneuploidy, cause embryo arrest, or result in implantation failure if transferred (67–70).

When gene expression patterns were analyzed in the blastocysts that did successfully form after SCD and WSD consumption, we observed a high number of genes that were differentially regulated between the 2 treatment groups. The genes upregulated in WSD blastocysts belonged to diverse biological processes associated with RNA binding, protein channel activity, pluripotency, and amino acid, as well as fatty acid metabolism. An upregulation of genes involved in mitochondrial translation was also detected in the blastocysts after WSD intake. These findings are supported by previous studies reporting that maternal high-fat diet increased mitochondrial membrane hyperpolarization and mitochondria DNA copy number to compensate for abnormal mitochondrial activity in murine oocytes and zygotes (7, 21, 71). Mitochondrial dysfunction could lead to apoptosis in subsequent preimplantation development as previously shown (72). In contrast, the genes that were significantly downregulated in WSD compared with SCD blastocysts were associated with processes important for early embryogenesis such as the cytokine/steroid response (73), cell proliferation (74), lineage specification (75), and differentiation of the placental-derived trophectoderm (76) or other extra embryonic lineages (77), as well as development of the mesoderm layer (78). Since all of these molecular pathways are critical for the peri-implantation period, we suspect that the WSD blastocysts may have had lower implantation rates if transferred, resulting in early embryo loss, or may have exhibited placental dysfunction after implantation, as shown with rhesus macaque females on a long-term WSD and obese women undergoing IVF treatment in previous studies (8, 79–81). Thus, despite selecting morphologically normal embryos with only bipolar divisions, there was still a substantial number of genes that were differentially regulated that potentially affect critical developmental functions.

In order to potentially improve embryo transfer success and pregnancy outcomes in obese patients, we suggest that a more in-depth assessment of the biological processes and signaling pathways revealed by our RNA-seq analysis be conducted on WSD embryos. Moreover, determining whether a similar proinflammatory follicular microenvironment, embryonic arrest, and differential gene expression, which is observed when the females are on a WSD for a longer period of time (i.e., more than a year of continual WSD consumption) and develop metabolic dysfunction, should also be determined. We note that there are already published findings of adverse effects from long-term WSD consumption on rhesus macaque ovarian and uterine structure and function (82, 83), but — to our knowledge — there are not yet studies on oocyte fertilization and preimplantation development during COS cycles; this is a current research focus of our group. The results of these studies will help determine the precise impact of WSD on infertility and its confounding effects on IVF success (8, 84). With the ultimate goal of reducing embryo loss, while increasing live birth rates, the findings from this study serve to advance our understanding of how maternal diet modulation affects embryogenesis and subsequent development in both obese and normal-weight women.

## Methods

### Cohort of rhesus macaque females and diet modulation.

A cohort of 10 regularly cycling female rhesus macaques of young reproductive age (5–6 years) and average body weight (5–7 kg) were chosen for this study. The rationale for selection of a cohort of 10 female rhesus macaques was based on a power analysis to detect statistical differences in a previous study that assessed the long-term effects of diet and hyperandrogenemia on reproduction (39). The females resided in the Oregon National Primate Research Center (ONPRC) Obese Resource, which provided animal care and research support. Each female initially underwent COS #1 after consuming a SCD (15% fat, 59% carbohydrate, 26% protein) since weaning (approximately 1 year of age). Their diet was switched to a high-fat WSD (36% fat, 46% carbohydrate, 18% protein) for 6–8 months before undergoing COS #2. The SCD is a fiber-balanced monkey diet from LabDiet that is a high-energy formulation to specifically support postpartum reproduction and is routinely used for experiments at ONPRC. It contains protein derivatives such as soybean and provides dietary fiber from more than 1 source. In contrast, the WSD has nearly half the fiber to that of the SCD and consists of refined sugar and corn-based derivatives. The fat content of the WSD is derived from animal/poultry fat.

### I.v. glucose tolerance testing (ivGTT) and body fat percentage.

Animals were fasted overnight and sedated with Telazol (5 mg/kg i.m. Tiletamine HCl/Zolazepam HCl, Fort Dodge Animal Health). If needed, additional anesthesia was accomplished with ketamine (3–10 mg/kg i.m., Abbott Laboratories). Once sedated, animals received an i.v. glucose bolus (50% dextrose solution) at a dose of 0.6 g/kg via the saphenous vein. Baseline blood samples were obtained prior to the infusion and at 1, 3, 5, 10, 20, 40, and 60 minutes after infusion. Glucose was measured immediately using FreeStyle Lite Glucose Monitor (Abbott Laboratories), and the remainder of the blood was kept in heparinized tubes on ice for insulin measurement. After centrifugation (1,000 x g for 10 minutes at 4ºC), samples were stored at –80°C until assayed. Insulin measurements were performed by the Endocrine Technologies Core (ETC) at the ONPRC using a chemiluminescence-based automatic clinical platform (Roche Diagnostics, Cobas e411).

Percent body fat for each animal was measured using dual-energy x-ray absorptiometry (DEXA; Hologic QDR Discovery A; Hologic Inc.). Total body scans were performed on the same day of the ivGTTs to minimize the number of sedations for each animal. Animals were positioned prone on the bed of the scanner, and QDR software (Hologic Inc.) was used to calculate percent body fat. All the metabolic parameter measurements were performed at 3 time points: 1–3 months after COS #1 when the females were consuming SCD, 4 months after WSD consumption, and 1 month after COS #2 (6–8 months after WSD consumption). To avoid potential transient interference with food consumption and weight changes associated with gonadotropin administration and laparoscopic follicle aspiration, the body weights were measured 1–3 months after COS rather than at the time of oocyte retrieval.

### Oocyte aspiration and processing.

Both COS cycles were performed as previously described (85). Briefly, exogenous gonadotropins were administered to stimulate the development of multiple ovarian follicles. A positive COS response was measured by serum E2 levels rising above 200 pg/mL on day 3 or day 4 of the COS protocol. Female rhesus macaques were anesthetized for laparoscopic follicular aspirations 36 hours after the administration of human chorionic gonadotropin (hCG) to induce events necessary for the reinitiation of meiosis. Two individual follicle aspirates per ovary were collected manually with a low dead space 3 mL syringe and 22 gauge × 1.5 inch needle (Ulticare) for each aspirate. Individual follicle aspirates were centrifuged at room temperature for 30 seconds at 1000*g* to separate the FF from the cumulus-oocyte complex (COC) and the GCs. The COCs were then examined for presence of oocytes under a stereomicroscope by dilution with Tyrode’s albumin lactate pyruvate (TALP) HEPES buffer. They were then denuded by gentle micropipetting in TALP buffer containing 0.3% BSA (MilliporeSigma). Each oocyte from an individual aspirate was placed in separate 100 μL TALP complete drops in a preequilibrated IVF dish covered by mineral oil (Sage). The FF and CCs were frozen down separately for further analysis. The rest of the follicular aspirates were collected in bulk using vacuum suction in TALP-HEPES buffer with 0.3% BSA and 1% heparin sodium salt solution at 37°C to obtain the remaining COCs. Oocytes from the bulk aspirates were denuded by gentle micropipetting in TALP-HEPES buffer containing 0.3% BSA and 3% hyaluronidase (MilliporeSigma). These oocytes were grouped according to their developmental stage and placed in 100 μL TALP complete drops in a preequilibrated IVF dish covered by mineral oil (Sage).

### IVF and embryo culture.

Fresh semen from 3 adult male rhesus monkeys of average age (9.4 ± 1.5 years old) and proven fertility was used for IVF throughout this project. Semen was collected from 1 of the 3 males the same day as oocyte retrieval for conventional IVF. In brief, seminal plasma was removed as previously described (86) and used for IVF at a final concentration of 2 × 10^6^ sperm/mL in TALP-complete medium. IVF was performed the evening of the collection. Sperm was treated with cyclic adenosine monophosphate (cAMP; 5 mg/mL) and caffeine (2 mg/mL) 15 minutes before fertilization to induce hyperactivation and increase fertilization potential. In each well containing oocytes, 1 μL of the activated sperm was added. IVF dishes were incubated at 5% CO_2_ and 37°C overnight.

After IVF for 14–16 hours, oocytes were stripped of excess sperm by pipetting and visually assessed for fertilization (i.e., the presence of 2 pronuclei and/or 2 polar bodies). Confirmed zygotes were individually transferred to custom Eeva 12-well polystyrene petri dishes (Progyny Inc.) for TLM and cultured in 100 μL of one-step commercial medium supplemented with 10% serum protein (LifeGlobal) under mineral oil (Sage) at 37°C with 6% CO_2_, 5% O_2_, and 89% N_2_. The remaining zygotes were transferred to a preequilibrated 10-well IVF dish and cultured in the same medium and incubator as the TLM dish. Embryo development was individually tracked through day 8 after IVF. Medium was changed at day 3 after IVF, and the embryos were left to continue developing to the blastocyst stage up until day 8. Arrested (preblastocyst stage) embryos and blastocyst development outcomes were recorded. Fertilization rate was calculated as the number of zygotes after IVF/number of mature MII oocytes × 100. Blastocyst formation rate was calculated as the number of blastocysts formed/number of cleaved embryos × 100.

### Time-lapse monitoring.

Individual confirmed zygotes transferred to a time-lapse monitoring (TLM) dish (*n* = 12) were monitored with an Eeva dark-field 2.2.1 time-lapse microscope system (Progyny Inc.) as previously described (87). Zygotes from 6 females from COS #1 and 9 females from COS #2 underwent TLM analysis. The Eeva TLM systems were composed of an inverted microscope with a 10× Olympus objective, auto-focus, and 5-megapixel CMOS digital camera placed inside a tri-gas incubator (Panasonic Healthcare). The embryos were imaged every 5 minutes with a 0.6 second exposure time until developmental arrest or up to 8 days if they progressed to the blastocyst stage. Each image was time stamped with a frame number, and all images were compiled into an AVI movie using FIJI software. The time intervals between the appearance of a cleavage furrow to the end of the first cytokinesis, the beginning of the second mitotic division, and the start of the third mitotic division were manually recorded and represented as an average. Other morphological features such as cellular fragmentation and asymmetrical/multipolar division were also examined and recorded for each embryo. A total of 174 TLM videos after SCD and after WSD intake were analyzed by 5 independent observers.

### Cytokine and steroid analysis of FF.

The FF from 2 individual aspirates collected from each female rhesus macaque were pooled together for each COS. These pooled FF samples were analyzed for concentrations of 29 cytokines and 7 steroids. Analyses of steroid hormone levels was performed in the ETC at ONPRC by LC-MS/MS on a Shimadzu Nexera-LCMS-8050 using a previously described method (65). According to ONPRC ETC records, accuracies range from 87.9% to 103.0% and intraassay coefficient of variation (CVs ) are < 4% for the steroid assays performed. Cytokine levels were determined using a rhesus macaque 29-plex cytokine panel (Thermo Fisher Scientific) following the manufacturer’s instructions. Concentrations of each cytokine were calculated from a standard control curve. Samples were analyzed on a Milliplex Analyzer (MilliporeSigma) bead sorter with XPonent Software version 3.1 (Luminex). Data were calculated using Milliplex Analyst software version 5.1 (MilliporeSigma). Intraassay CVs for cytokine assessment through the ONPRC ETC 29-plex panel is < 15%. The list of all the cytokines and steroids measured, along with their individual CVs, are included in Supplemental Tables 1 and 2, respectively.

### RNA-seq.

Blastocysts were collected from COS #1 (*n* = 138) and COS #2 (*n* = 77) and incubated in EmbryoMax Acidic Tyrode’s Solution (MilliporeSigma) for approximately 30 seconds for removal of the zona pellucida. These zona free blastocysts were then transferred to the extraction buffer of the ARCTURUS PicoPure RNA Isolation Kit (Thermo Fisher Scientific, KIT0204) and frozen at –80°C until RNA isolation. From all of the blastocysts, 33 were chosen for RNA-seq analysis to equalize the number of embryos from both diet groups. To minimize variability between treatment groups due to abnormal cytokinesis, only blastocysts that exhibited bipolar divisions were sequenced as shown in Supplemental Table 3. RNA was extracted from the blastocysts and cDNA prepared using the SMART-Seq v4 Ultra Low Input RNA Kit for sequencing (TakaraBio), and the amplified cDNA was purified using the Agencourt AMPure XP Kit (Beckman Coulter), both according to manufacturer instructions. cDNA was then sheared to approximately 250 bp in length using a Covaris M220 sonicator. Sheared cDNA was resuspended in Tru-seq Resuspension Buffer, and libraries were prepared using a Tru-Seq Nano kit (Illumina) according to the manufacturer’s instructions, except that 16 cycles of amplification were performed to account for the low-input samples. Fragment size was measured using a Fragment Analyzer 5200 and samples quantified with quantitative PCR (qPCR) and pooled at equimolar concentration. Multiplexed samples were sequenced across 7 lanes of a single-read, 75-cycle run on an Illumina HiSeq 4000 sequencer. The sequencing data were demultiplexed using Illumina’s bcl2fastq software, and sample quality was assessed with FastQC (v 0.11.8), followed by trimming of low-quality bases and adapter sequences with Trimmomatic (v 0.39). Trimmed sequences were aligned via STAR (version 2.7.0) to the most recent rhesus macaque reference genome from Ensembl (Mmul_10), and gene counts were obtained by specifying the “quantMode GeneCounts” parameter of STAR, along with the Mmul_10.99 Ensembl annotation gtf file. Two outliers (one from the SCD group and another from the WSD group) had lower gene counts than expected, which was likely due to DNA contamination, and they were removed from further analyses. Differential expression between groups was performed with edgeR (version 0.28.0) using the “QLFTest” option. The Enrichr and G-profiler online tools were used for molecular pathway and GO assessment. All RNA-seq data has been deposited in the genome expression omnibus (GEO) database, accession no. GSE162817 (https://www.ncbi.nlm.nih.gov/geo/query/acc.cgi?acc=GSE162817).

### Statistics.

Repeated measures one-way ANOVA with multiple comparisons was performed to assess any significant differences in body weight, body fat percentage, HOMA-IR, glucose AUC, and insulin AUC. Paired *t* test was performed to assess significant differences in cytokine or steroid levels in the FF and the number of blastocysts formed before and after WSD consumption. The logistic mixed effects regression models with random intercept was used to account for intrafemale correlation to the odds ratio of obtaining oocytes at different stages of maturity, any significant differences in the rates of fertilization, cleavage, and blastocyst formation, as well as the incidence of cellular fragmentation and/or multipolar divisions between the 2 groups. RNA-seq data *P* values were adjusted for multiple comparisons with the Benjamini-Hochberg method.

### Study approval.

All protocols involving animals were approved by the ONPRC IACUC and conducted in accordance with the *Guide for the Care and Use of Laboratory Animals* (National Academies Press, 2011). The housing and general care of rhesus macaques (*Macaca mulatta*) was previously described (88).

## Author contributions

SR, PK, SLC, and JDH designed the study, performed experiments, analyzed data, and wrote the manuscript. AYT performed the oocyte collection and monitored the preimplantation development for COS #1. CAM, DLT, and PK performed animal care and metabolic studies. MJM helped with coordinating and scheduling for COS cycles. SR, MJM, NRT, DW, and SLC were each an independent observer for TLM analysis. All authors were involved in editing the manuscript.

## Supplementary Material

Supplemental data

Supplemental Video 1

Supplemental Video 2

## Version 1 02/22/2021

Electronic publication
