# Supplementary material for: Short-term Western-style diet negatively impacts reproductive outcomes in primates
Source: JCI Insight. 2021 Feb 22;6(4):e138312. doi: 10.1172/jci.insight.138312 (PMC7934943; doi:10.1172/jci.insight.138312)
Supplement: Supplemental data [file jciinsight-6-138312-s048.pdf]

Supplementary Figure 1. Quality control assessment of the RNA-seq data from blastocysts. **(a)** Bar graph showing the total gene counts per sample after alignment. Note that two samples, one from each COS, exhibited a reduced number of total genes highlighted in pink. **(b)** A Euclidean distance clustering map demonstrated that these two outliers clustered together away from the other samples and were likely contaminated with DNA.

Supplementary Table 1. Assay metrics for the cytokine analysis. The concentration of 29 cytokines was analyzed in the follicular fluid samples of the female rhesus macaques post-SCD and post-WSD with an automated multiplex ELISA system (Luminex 29-plex). Intra-assay co-efficient of variation (CV) has been provided for each cytokine analyzed in the table below.

Target Cytokine	Intra-Assay CV (%)
FGF Basic	2.64
IL1B	3.13
G-CSF	3.57
IL10	3.86
IL6	3.00
IL12	4.62
RANTES	11.27
Eotaxin	14.23
IL17	8.86
MIP1A	2.79
GMCSF	5.72
MIP1B	3.98
MCP1	4.86
IL15	10.05
EGF	3.81
IL5	9.48
HGF	3.59
VEGF	11.06
IFNG	2.88
MDC	4.63
ITAC	10.21
MIF	0.93
IL1RA	4.78
TNFA	7.22
IL2	8.23
IP10	4.59
MIG	0.00
IL4	4.44
IL8	2.94

Supplementary Table 2. Assay metrics for the steroid analysis (LC/MS). The concentration of seven steroids were assessed in the follicular fluid samples of the female rhesus macaques post-SCD and post-WSD by LC/MS. Intra-assay co-efficient of variation (CV) and accuracy of the assay has been provided for each steroid analyzed in the table below.

**Steroid Hormone**	**Accuracy (%)**	**Intra-assay CV (%)**
Cortisol	93.9	1.5
Cortisone	94.0	2.1
Testosterone	94.4	4.0
Estradiol	101.1	2.8
Estrone	99.2	2.4
Progesterone	87.9	1.8
Androstenedione	103.0	3.5

Supplementary Table 3. Distribution of blastocyst samples for RNA-seq from COS #1 and COS #2. RNA-Seq analysis was performed on 15 samples from COS #1 and 18 samples from COS #2. These samples were grouped based on occurrence of fragmentation and multipolar divisions during preimplantation development. The table also indicates the females to which the blastocysts can be attributed.

Sample Name	Embryo Information	Female Number
COS_1_1	Fragmented, non-multipolar	1
COS_1_2	Fragmented, non-multipolar	2
COS_1_3	Fragmented, non-multipolar	2
COS_1_4	Fragmented, non-multipolar	3
COS_1_5	Fragmented, non-multipolar	4
COS_1_6	Fragmented, non-multipolar	4
COS_1_7	Fragmented, non-multipolar	5
COS_1_8	Non-fragmented, non-multipolar	1
COS_1_9	Non-fragmented, non-multipolar	1
COS_1_10	Non-fragmented, non-multipolar	2
COS_1_11	Non-fragmented, non-multipolar	2
COS_1_12	Non-fragmented, non-multipolar	3
COS_1_13	Non-fragmented, non-multipolar	5
COS_1_14	Non-fragmented, non-multipolar	5
COS_1_15	Non-fragmented, non-multipolar	5
COS_2_1	Fragmented, non-multipolar	1
COS_2_2	Fragmented, non-multipolar	2
COS_2_3	Fragmented, non-multipolar	2
COS_2_4	Fragmented, non-multipolar	3
COS_2_5	Fragmented, non-multipolar	3
COS_2_6	Fragmented, non-multipolar	4
COS_2_7	Fragmented, non-multipolar	4
COS_2_18	Fragmented, non-multipolar	7
COS_2_8	Fragmented, non-multipolar	6
COS_2_9	Non-fragmented, non-multipolar	1
COS_2_10	Non-fragmented, non-multipolar	1
COS_2_11	Non-fragmented, non-multipolar	2
COS_2_12	Non-fragmented, non-multipolar	2
COS_2_13	Non-fragmented, non-multipolar	3
COS_2_14	Non-fragmented, non-multipolar	5
COS_2_15	Non-fragmented, non-multipolar	5
COS_2_16	Non-fragmented, non-multipolar	5
COS_2_17	Non-fragmented, non-multipolar	5
